# Therapy preferences in melanoma treatment—Willingness to pay and preference of quality versus length of life of patients, physicians, healthy individuals and physicians with oncological disease

**DOI:** 10.1002/cam4.3191

**Published:** 2020-07-10

**Authors:** Julia Weiss, Michael Constantin Kirchberger, Lucie Heinzerling

**Affiliations:** ^1^ Friedrich‐Alexander‐University Erlangen‐Nürnberg (FAU) Erlangen Germany; ^2^ Department of Dermatology University Hospital Erlangen Friedrich‐Alexander‐University Erlangen‐Nürnberg (FAU) Erlangen Germany

**Keywords:** cancer management, clinical guidelines, immunology, quality of life

## Abstract

**Background:**

In recent years, monoclonal antibodies such as ipilimumab, nivolumab, and pembrolizumab have made a significant impact on the treatment of advanced melanoma. Combination of immune checkpoint inhibitors leads to improved survival and response rates of 58%‐61% as compared to monotherapy (36%‐44%). However, the price for the better response rates is also a higher frequency of severe adverse events (59%) as compared to monotherapy (17%‐21%). This study examines attitudes towards melanoma therapy options of physicians, healthy individuals, melanoma patients, and physicians with oncological disease, their willingness to pay, and preference of quality versus length of life.

**Methods:**

After obtaining ethical approval and informed consent surveys were conducted in 111 participants divided into four groups: melanoma patients (n = 30), healthy individuals as controls (n = 30), physicians (n = 27), and physicians with oncological disease (n = 24). Statistical analyses were conducted using SPSS statistics (version 25, IBM), applying the Pearson´s chi‐squared test, Spearman correlation coefficient, Wilcoxon‐Mann‐Whitney test, and Kruskal‐Wallis test.

**Results:**

Life prolongation is more valued by melanoma patients and physicians with oncological disease compared to healthy controls and healthy physicians. In total, 30% of melanoma patients opt for a life prolonging therapy in all cases, even if this life prolongation is only marginal. Physicians are the strongest proponents of combination immunotherapy.

**Conclusion:**

The valuation of the different treatment options differs in the four study groups with affected people valuing life prolongation much more. The individual value of cancer therapies is high, but differs from the societal standpoint.

## Graphical abstract

Valuation of different melanoma treatment options differs signficantly among different study groups with affected people valuing life prolongation much more. The individual value of cancer therapy is high, but differs from the societal standpoint.

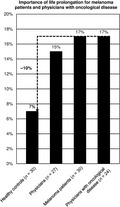

## 1 INTRODUCTION

Checkpoint inhibitor therapy has shown efficacy in various cancer entities. In metastatic melanoma both monotherapy with anti–programmed cell death‐1 protein (anti–PD‐1) antibodies (nivolumab, pembrolizumab)^1^, ^2^ and combination therapy with anti–PD‐1 plus anti–cytotoxic T‐lymphocyte antigen‐4 (anti–CTLA‐4; ipilimumab)^3^ have increased overall survival rates.^4^ Median overall survival under the therapy with nivolumab with 16.8 months is comparable to pembrolizumab with 20 months in two different trials in metastatic melanoma patients.^1^, ^2^ The combination therapy is more effective than monotherapy with significantly longer median progression‐free survival of 11.5 months compared to 6.9 months for nivolumab in the Checkmate 067 trial.^5^ Although median overall survival had not been reached yet in this study, first‐line combination therapy was estimated to offer an additional 14.4‐36 months of life time compared to first‐line CTLA‐4 or anti–PD‐1 alone.^5^, ^6^

While combination therapy has higher response rates compared to anti–PD‐1 therapy (58%‐61%^7^, ^8^ vs 36%‐44%^7^, ^9^), it has also significant toxicity with treatment‐related adverse events grade 3 or 4 in 59%,^3^ compared to 17%‐21% for monotherapy. Immune checkpoint inhibitors are capable of inducing a plethora of adverse events. Adverse events typically affect the skin, gastrointestinal, hepatic, and endocrine systems.^10^ While symptoms of adverse events can usually be managed, permanent sequelae and even fatalities have occurred.^11^, ^12^

The unprecedented costs represent one further drawback of these novel drugs, with around €29'226 per cycle for nivolumab and €73'338 per cycle for the combination therapy (for the drug only, Information from the pharmacy of the University Hospital Erlangen).^13^ This raises questions on the cost‐effectiveness since physicians must decide between treatment options for each individual patient and weigh them against each other regarding treatment‐related adverse events, life prolongation, and also quality of life. This should include individual factors and patients’ preferences.^14^

This questionnaire‐based study aimed to investigate the attitudes of melanoma patients, healthy individuals, physicians, and physicians with oncological disease toward immune checkpoint inhibitors in the context of adverse events, life prolongation and quality of life during immunotherapy. In particular, we tried to evaluate preferences toward combination immunotherapy with approved dosing, reduced dosing (ipilimumab 1 mg/kg of bodyweight and pembrolizumab 2 mg/kg of bodyweight or nivolumab 3 mg/kg of bodyweight) and anti–PD‐1 monotherapy.

## 2 PATIENTS, MATERIALS, AND METHODS

Within this study, we developed a questionnaire in German and pretested it in a group of participants to identify any misunderstanding and revised the questionnaire accordingly. Once finalized, we distributed the questionnaire among four groups: melanoma patients, healthy individuals as controls, physicians, and physicians with oncological disease.

Melanoma patients were recruited from the outpatient and inpatient Department of Dermatology of the University Hospital Erlangen. Only patients who were—according to their attending physician—in a good physical and mental condition could participate in the study. Healthy individuals were defined as absence of cancer with similar baseline characteristics comparable to the patients’ group; however, their current state of health may be limited by other general diseases. Similar baseline characteristics encompassed a comparable age of study participants, family status, parenthood, religious beliefs, and educational background. Table 1 documents the baseline characteristics of the different groups.

**TABLE 1:** Characteristics of the study groups

	Healthy individuals n = 30	Physicians n = 27	Melanoma patients n = 30	Physicians with oncological disease n = 24
Age				
Range	23‐71	26‐67	30‐81	28‐77
Median	62.5	37.5	59.5	57.5
Gender				
Male	40% (12)	59% (16)	63% (19)	58% (14)
Female	60% (18)	41% (11)	37% (11)	41% (10)
Family status				
Alone	17% (5)	23% (6)	20% (6)	13% (3)
With partner	57% (17)	38% (10)	57% (17)	42% (10)
With partner and child	20% (6)	38% (10)	23% (7)	42% (10)
Single parent	0% (0)	0% (0)	0% (0)	4% (1)
With others	7% (2)	0% (0)	0% (0)	0% (0)
Children				
Yes	62% (18)	54% (14)	77% (23)	79% (19)
No	38% (11)	46% (12)	23% (7)	21% (5)
Dependents				
Yes	19% (5)	52% (13)	21% (6)	45% (9)
No	81% (22)	48% (12)	79% (22)	55% (11)
Religious belief				
None	23% (7)	44% (11)	13% (4)	17% (4)
Little	13% (4)	28% (7)	20% (6)	25% (6)
Medium	23% (7)	20% (5)	40% (12)	21% (5)
High	30% (9)	8% (2)	27% (8)	29% (7)
Education				
None	7% (2)	0% (0)	3% (1)	0% (0)
Apprenticeship	41% (12)	0% (0)	45% (13)	0% (0)
Master/technical college degree	14% (4)	0% (0)	24% (7)	0% (0)
University degree	34% (10)	100% (27)	24% (7)	100% (24)
Employment				
Employee	33% (10)	81% (21)	45% (13)	58% (14)
Self‐employed	3% (1)	12% (3)	3% (1)	17% (4)
Other	60% (18)	0% (0)	52% (15)	25% (6)
Gross income per month				
Median	2000‐3500 Euros	5000+ Euros	1000‐2000 Euros	5000+ Euros
Modus	2000‐3500 Euros	5000+ Euros	1000‐3500 Euros	5000+ Euros

Physicians engaged in the Dermatology and Palliative Medicine Departments of the University Hospital Erlangen, the Nuremberg Hospital, and the Internal Medicine Department of the Neumarkt Hospital participated in the study. The recruitment of physicians with oncological disease was carried out via the outpatient clinic of the Department for Dermatology at the University Hospital Erlangen and several large cancer centers in Germany (Dresden, Tübingen, Kiel and Essen).

Approval from the ethics commission of the medical faculty of the “Friedrich‐Alexander Universität Erlangen‐Nürnberg” [EK_No. 40_12 Bc] was obtained.

The questionnaire (English version; shown in Data S2) encompassed questions on palliative care vs tumor therapy, allocation of financial resources, and prolongation of life plus adverse events vs no symptoms with a shorter life span. Most questions were designed with responses on a five‐point Likert scale from 0 to 4 (0—“I absolutely disagree,” 1—“I disagree,” 2—“I am undecided,” 3—“I agree,” and 4—“I absolutely agree”) or dichotomous for choices between different cancer therapy options. The decision had to be made between various therapy suggestions with differing levels of life prolongation and resulting adverse events.

Participants were asked about three different treatment options of melanoma therapy: the anti–PD‐1 monotherapy characterized by a moderate response rate, a moderate life prolongation and severe adverse events in 15% of cases. As an alternative, the combination therapy with higher response rates and life prolongation but also a twice as high rate of adverse events was presented to the study participants. The third option was palliative care which was presented as not life‐prolonging but symptom‐free. The four study groups had to evaluate different statements on these three treatment options and weigh up between life prolongation and toxicity vs quality of life. They had to decide which therapy they would prefer for themselves and which one they would recommend to others. In addition, they should try to determine the value of each therapy as their willingness to pay, usually predetermined by society, economic system, and individual circumstances.

Baseline data regarding their health status was collected for all participants. In order to verify the declared state of health, study participants were asked to indicate how high they estimate their overall life expectancy.

The participation in this study was voluntary for all respondents. Participants were informed at the beginning of the survey that the question of the present study was hypothetical, and all data would be analyzed and published anonymously. Patients were advised that participation in the study would not influence their medical therapy. In case that ambiguities arose when answering the questions, patients received assistance while filling out the questionnaire.

All questionnaires of the patients and the physicians of the Department of Dermatology in Erlangen were collected in drop‐off boxes in the outpatient department of Dermatology at the University Hospital Erlangen. The healthy control group, physicians practicing in other hospitals, and affected physicians who were cared for in several cancer centers in Germany were able to send the completed questionnaires back to the Department of Dermatology in prepaid envelopes.

### 2.1 Statistical analysis

The statistical analysis of the data was carried out using Microsoft Excel (Microsoft Corporation, 2016) and SPSS Statistics (IBM, version 25). If values were missing, the entire case was deleted from the data record. The Pearson´s chi‐squared test was used to compare non ordinal data such as children, faith, family status, and education. The effect level was interpreted by Cramer's V according to Cohen. The statistical significance was determined using Monte Carlo simulation. Correlations between ordinal data including income, health status, and life expectancy were illustrated using the Spearman correlation coefficient. Statistically significant correlations between nominal and ordinal data were determined with the Wilcoxon‐Mann‐Whitney test (evaluation of ordinal data and nominal data with two values) and the Kruskal‐Wallis test (evaluation of ordinal data and nominal data with more than two values). The two‐ tailed *t* test was used to determine whether two samples were statistically significantly different. The main statements of the Likert scales were calculated with the median value. The frequencies of the answers in the areas “I absolutely agree” and “I agree” were added up. The same procedure was applied in the areas “I absolutely disagree” and “I disagree.” All metric data such as current health status and estimated life expectancy were computed using the mean value. A value of *P* ≤ .05 was considered statistically significant. Due to excluded data, percentages may not add up to 100%.

## 3 RESULTS

Overall 120 questionnaires returned to the melanoma cancer centre of the University Hospital Erlangen; 111 questionnaires (n = 93%) could be analyzed for this study since the other nine questionnaires were returned empty. The study groups showed comparable sizes with 24‐30 respondents each. The average age of the participants was between 57.5 and 62.5 years, apart from the physicians who had a lower average age of 37.5 years. Overall, 45% of the respondents were female. Baseline characteristics like age, gender, family status, children, faith, education, and economic status were collected at the end of the survey. For detailed information on the composition of the study groups, please refer to Table 1.

The estimated life expectancy was highest among the healthy individuals with a mean of 85 years. For physicians and physicians with oncological disease, life expectancy was 80 years; melanoma patients estimated their life expectancy to be the lowest at a mean of 78 years. Scenarios were carried out for all study groups confronting participants with choices to live without any health problems until the end of their lives but would have to sacrifice several life years for this. On average, all respondents would sacrifice a mean of 4 years of life (range 2‐5 years) in order to life without any health problems.

For all groups, age correlated with the question of how many years of life study participants were willing to sacrifice (Spearman correlation coefficient *r* = −.255; *P*‐value = .0078; q. 3). Among younger participants, the willingness to sacrifice life years in order to maintain full health was higher as compared to older participants. The current state of health also correlated with the willingness to give up lifetime for life quality (Spearman correlation coefficient *r* = .259; *P*‐value = .0069; q. 3), in detail, the better their current state of health, the more years of life participants were willing to sacrifice.

### 3.1 Importance of life extension in immunotherapy

**FIGURE 1:**
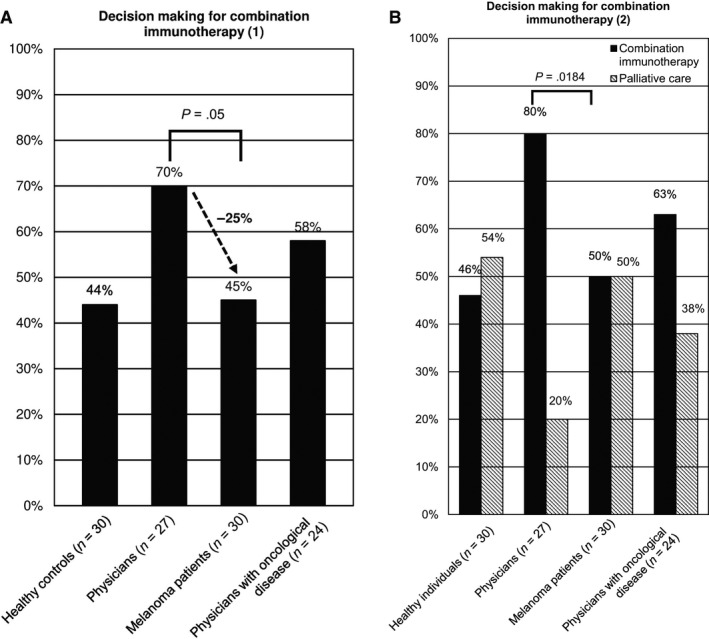
(A) Decision making for combination immunotherapy (1). With 70%, physicians are the strongest proponents of combination immunotherapy. In comparison, 45% of patients chose combination immunotherapy, which corresponds to a reduction of 25%. (B) Decision making for combination immunotherapy (2). About 80% of the physicians preferred combination immunotherapy compared to melanoma patients with 50% if the second therapy option was palliative care

Participants had the choice between combination immunotherapy, which would be accompanied by severe adverse events, and prolongation of the life expectancy of 19 months and monotherapy with fewer adverse events and consequently a lower life prolongation with only 9 months (q. 4). Whereas 58% of physicians with oncological disease opted for longer life, melanoma patients chose this option in only 45% (*t* test; *P*‐value = .0543). Healthy physicians, in contrast, were the strongest proponents of combination immunotherapy with 70% (Figure 1A; q. 4).

**FIGURE 2:**
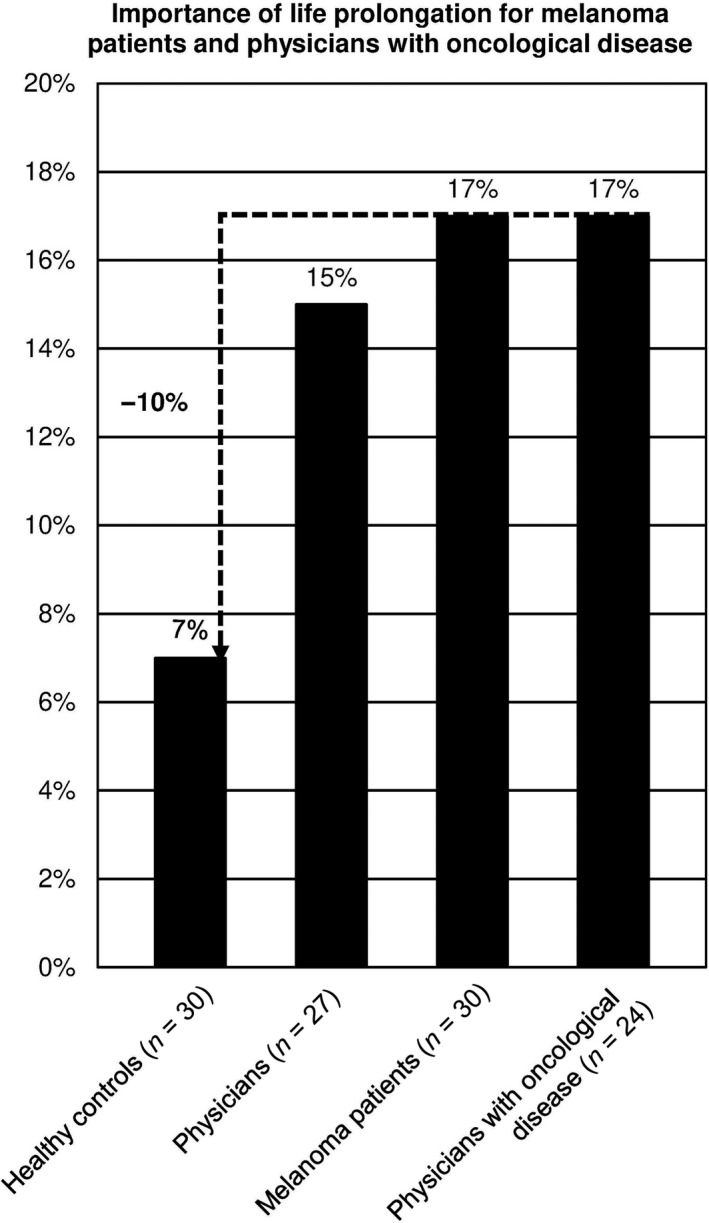
Importance of life prolongation for melanoma patients and physicians with oncological disease. About 17% of the patients and physicians with oncological disease opted for the combination immunotherapy, even if a marginal life extension of 1 month would be achieved

Even if the life extension due to immunotherapy was only 1 month, 17% of melanoma patients and physicians with oncological disease opted for combination immunotherapy with more frequent serious adverse events (Figure 2; q. 6).

The therapy with severe adverse events and 24 months life extension was preferred by 80% (*t* test; *P*‐value = .0184) of the physicians and by 63% of the physicians with oncological disease (Figure 1B; q. 5). Participants in all four study groups opted more often for a life‐prolonging therapy if the respondents had children (Pearson´s chi‐squared test 5.041^2^ (1); *P*‐value = .0248; Phi 0.219; q. 5). Moreover, the better the respondents' economic status, the more likely they were to opt for a life‐prolonging therapy (Wilcoxon‐Mann‐Whitney test; *P*‐value = .0419; q. 5). The study groups of physicians and physicians with oncological disease were much more willing to accept adverse events for a prolongation of life. The stronger the religious faith among the physicians with oncological disease, the more frequently they decided for the therapy form of palliative care (Pearson´s chi‐squared test 9.145^2^ (3); *P*‐value = .0180; Cramer‐V 0.631; q. 7).

A third of the patients (30%) opted for a life‐prolonging therapy, even if this life‐prolongation was very low. About 67% of the healthy individuals, 60% of the physicians, and 70% of the physicians with oncological disease voted against a therapy with the prospect of a low life prolongation. The group of physicians with oncological disease was influenced in this decision by whether someone was dependent on them, such as a person in need of care (Wilcoxon‐Mann‐Whitney test; *P*‐value = .0465; q. 8).

In the role of the treating physician, 63% of the physicians, 50% of the physicians with oncological disease, and 47% of the melanoma patients recommended immunotherapy despite a possible high rate of adverse events (q. 23). Around 33% of the healthy individuals were undecided in this case. It was statistically dependent on whether the study participants in the role of the treating physician would recommend immunotherapy to their patients if they themselves were willing to undergo a therapy with serious adverse events (Spearman correlation coefficient *r* = −.412; *P*‐value ≤ .0001; q. 23). However, if the prolongation of life using immunotherapy was limited, all study participants were against recommending immunotherapy for their patients (q. 24). Recommendations also depended on the patient's willingness to undergo an immunotherapy with a short life span (Spearman correlation coefficient *r* = .341; *P*‐value = .0003; q. 24).

### 3.2 Factors influencing decision making

For all four groups, the recommendation of their treating physician as well as the advice of family and friends was crucial for the decision on treatment options. Among the healthy individuals, 43% stated that they were mainly influenced by the advice of their family and friends (q. 10). And 40% of melanoma patients and physicians agreed with this. Physicians with oncological disease were least influenced in their decision making by the advice of their family (25%). This fact shows a statistical correlation with the characteristic whether the physicians with oncological disease had children (Wilcoxon‐Mann‐Whitney test; *P*‐value = .0240; q. 10). Even more important than the advice of family and friends was the advice of the treating physician (q. 14). Half of the physicians with oncological disease (50%) agreed with this question. Among the healthy individuals it was 53%, among the physicians it was 59%, and among the melanoma patients it was 67%.

### 3.3 Value of longer therapy intervals for a better quality of life

**FIGURE 3:**
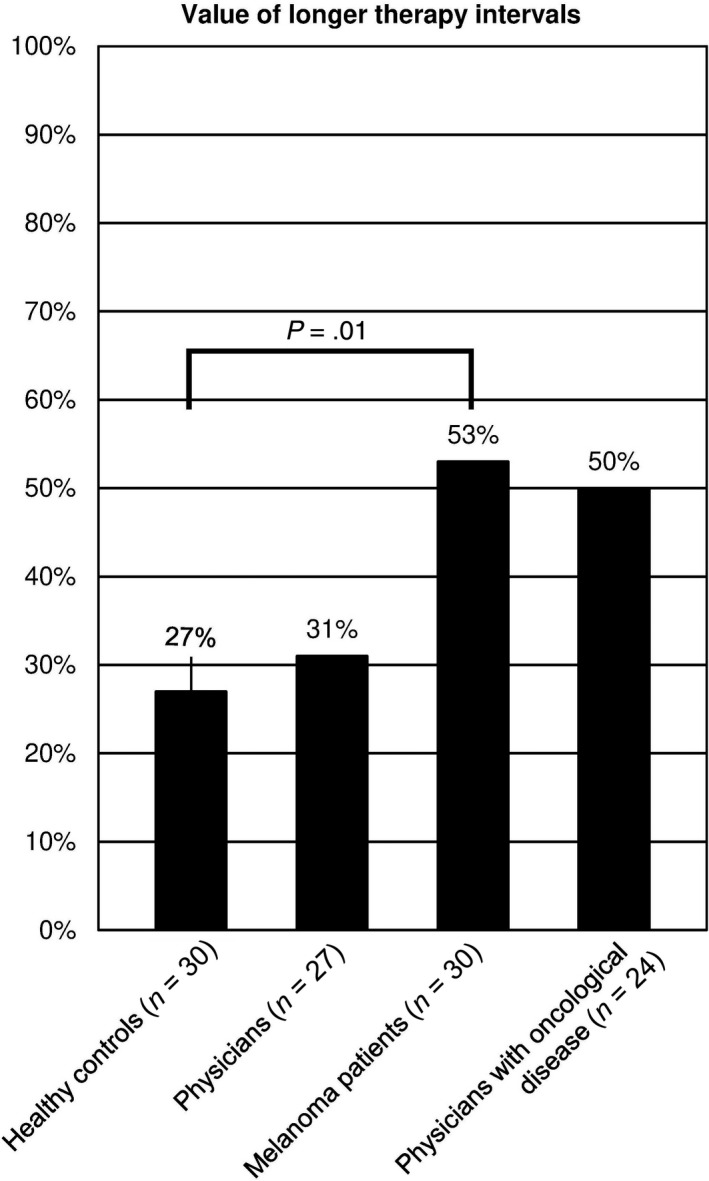
Value of longer therapy intervals. Compared to the healthy control group, melanoma patients were less willing to pay money for an extension of therapy intervals from 2 to 3 weeks

Around 96% of the physicians with oncological disease, 93% of the healthy individuals, 92% of the physicians, and 83% of the melanoma patients agreed that they rather received immunotherapy every 3 weeks instead of every 2 weeks for the same effect (q. 13). Most patients (53%; *t* test; *P*‐value = .0122) were not willing to pay money for longer therapy intervals, which was statistically dependent on the level of patients’ monthly gross income (Spearman correlation coefficient *r* = .423; *P*‐value = .0199; q. 17). The group of physicians with oncological disease was divided in this respect: about 50% were not willing to pay money for an extension of the therapy intervals; 21% on the other hand paid €1’000 for it (Figure 3, q. 17).

A different situation arose in the group of physicians: 19% were willing to pay €1’000 for a longer therapy interval; the same number even paid more than €1’000. The highest value a physician was willing to pay was €10’000. There was a statistically significant connection between the willingness to pay for longer therapy intervals and the economic power of physicians (Spearman correlation coefficient *r* = −.425; *P*‐value = .0385; q. 17).

### 3.4 Choice of early palliative care

If there was no prospect of healing, 70% of the healthy individuals opted for palliative care, followed by the physicians with 59%, physicians with oncological disease with 58%, and melanoma patients with 57% (q. 11). The more years of life melanoma patients were willing to sacrifice for a life free of complaints, the more likely they were to opt for palliative care (Spearman correlation coefficient *r* = −.351; *P*‐value = .0616; q. 11).

All study groups, with exception of the physicians with oncological disease, preferred palliative care if their current state of health was bad due to cancer disease (q. 9). This opinion could also be transferred to the thought construct in which the study participants should put themselves in the role of the treating physician. If there was no prospect of a cure, 83% of healthy individuals, 63% of physicians with oncological disease, 59% of physicians, and 53% of melanoma patients tried to improve their patient's quality of life i.e. by palliative care as opposed to tumor specific therapy in the hypothetical role of the treating physician (q. 31). If the study participants would rather want to improve the quality of life of their patients in the role of the treating physician, they would also—if they were suffering from cancer themselves—prefer to make the best of their remaining life time rather than undergo a stressful cancer therapy (Spearman correlation coefficient *r* = .497; *P*‐value ≤ .0001; q. 31). Already at the time of the cancer diagnosis, most of all groups pointed out the possibility of palliative care (q. 29). There was a statistical correlation between the recommendation for palliative care and their current health status of all study participants (Spearman correlation coefficient *r* = .194; *P*‐value = .0435; q. 25). A relation could also be established with the question of how many years of life they were willing to sacrifice for a life without any health problems (Spearman correlation coefficient *r* = .371; *P*‐value = .0008; q. 25).

### 3.5 Health economic aspect

From a health economic point of view, the new immunotherapies are associated with a high burden of cost. The expenditure for one treatment cycle is up to € 73'338 per patient for the medication only (Information from the pharmacy of the University Hospital Erlangen),^13^ whereas the annual costs for palliative care are significantly lower.

**FIGURE 4:**
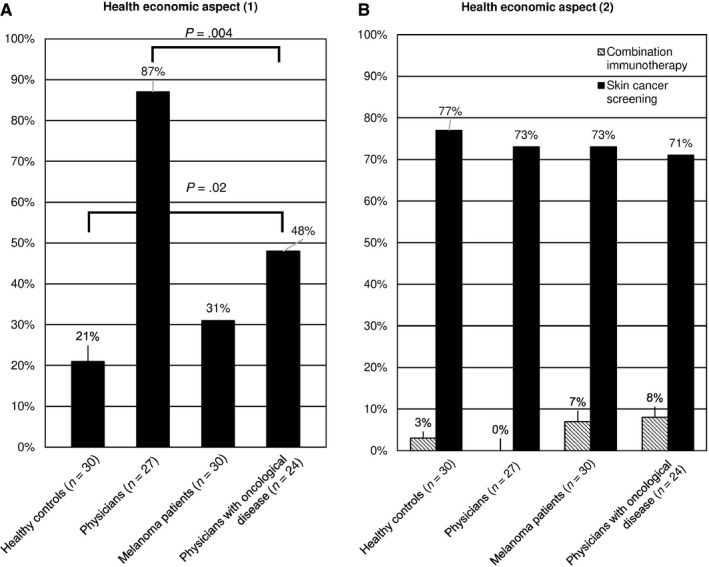
(A) Health economic aspect (1). From a societal point of view, 87% of physicians and 48% of physicians with oncological disease invested the sum of €1.5 million in combination immunotherapy. (B) Health economic aspect (2). From a social point of view, the majority of all groups opted for an investment of €150'000 in prevention measures instead of the combination of immunotherapy. All *P*‐values given in the figures were determined using a two‐tailed statistical *t* test

Against this background, the study participants should make the following decision: they could allocate €1.5 million from the health fund either to finance palliative care of 306 patients with an improved quality of life in the last month or to treat 10 patients with immunotherapy and enable them to survive an average of 21 months longer (q. 20). About 69% of the melanoma patients opted for palliative therapy. The group of physicians with oncological disease were devided in their opinion: while 52% opted for palliative care, 48% (*t* test; *P*‐value = .0209) would invest the money in immunotherapy. About 87% (*t* test; *P*‐value = .0042) of the physicians favored immunotherapy over palliative care in their investment (Figure 4A; q. 20).

Another scenario was presented to the study participants in this context. If they received €150’000 from the health fund and could use the money for palliative care, immunotherapy, or skin cancer screening for early cancer detection, all favored skin cancer screening; in detail 77% of the healthy individuals, 73% of the patients, 73% of the physicians, and 71% of the physicians with oncological disease (Figure 4B; q. 21).

A completely contrary view emerged under the condition that the study participants should put themselves in the position of the treating physician. They were asked whether they would spend money for immunotherapy or whether they would invest into skin cancer screening for the early detection of skin cancer (q. 30). Half of the melanoma patients did not hold back in the role of the treating physician the recommendation of the immunotherapy, likewise 54% of the physicians and 63% of the physicians with oncological disease. In the healthy individuals’ group, 40% were reluctant to recommend immunotherapy, 30% were undecided, and 30% recommended immunotherapy regardless of the costs incurred. The older the study participants, the more likely that they would spend money for immunotherapy (Spearman correlation coefficient *r* = .324; *P*‐value = .0006; q. 30).

All tested correlations that were not statistically significant, are listed in the Data S3.

## 4 DISCUSSION

It was difficult to determine the individual value of immunotherapies. Krammer et al found that patients are willing to pay money for life‐prolonging cancer therapies per se.^15^ Participants in this study are only to a certain extent willing to pay money that is, for an improvement in therapy conditions, such as an extension of therapy intervals from 2 to 3 weeks. This fact has been proven to be related to the patients' monthly gross income. Another reason for this may be that patients feel better cared for when they visit the hospital for therapy at shorter intervals.

However, melanoma patients in this study were not willing to accept a life‐prolonging therapy at any price because the acceptance of such a therapy regardless of the adverse events was rather low. This fact was supported by the argument that patients were willing to sacrifice 4 years of their lifetime in order to live completely symptom‐free until the end of their lives. Ultimately, life extension played a very important role for patients, but not regardless of the quality of life in the remaining time. The reason why melanoma patients were less willing to accept side effects to prolong life cannot be clearly defined. Factors postulated in this study—in particular age, gender, family status, children, religious belief, state of health, and economic power—showed no association to this question. However, in addition to the factors mentioned above, other parameters could also play a decisive role. The mental state of the melanoma patient as well as a possible depression as a concomitant symptom of the cancer disease should be mentioned.^16^

Adverse events should not just be seen as a direct result of immunotherapy, such as fatigue, colitis, pancreatitis and various others.^17^ As Levy et al describe, living with melanoma under immunotherapy could be compared to living in a phase of uncertainty, like playing a Russian roulette.^18^ Particularly noteworthy was the fact that all grades of adverse events had an impact on the quality of life of the patient, besides the less common adverse events.^19^

The discussion about the use of financial resources for immunotherapy, palliative care, or alternatively for other sectors such as prevention or cancer research is controversial. Most melanoma patients and healthy individuals invest the money in palliative care, whereas the physicians consider an investment in immunotherapy more reasonable. Interestingly, the group of physicians with oncological disease is rather undecided: palliative care and immunotherapy are preferred in almost equal parts. This suggests that the cancer disease of the physicians significantly influences their decision‐making toward a melanoma patient's view.

If, however, one proceeds one step further in this scenario and offers a selection of preventive measures for the early detection of skin cancer as a further investment option, all four study groups decide to invest in early detection measures. In this way, costs for expensive immunotherapies and complex palliative care can be reduced to a certain extent.

Against the background that immunotherapies mean substantial costs for our health care system, Curl demands that it is above all in the hands of society to deal consciously with the monetary resources of the health care system and to always critically question the cost‐benefit ratio of the therapies applied.^20^ In countries without a general health insurance another potential parameter could be payer/insurance status of the patient.

### 4.1 Limitations of the study

Due to the small sample size, the statements made in this study cannot be generalized. The study offers an insight into the different opinions of the four study groups. The present study is a qualitative study. The given questionnaires were not validated according to the statistical methods and not tested for internal consistency before application.

## 5 CONCLUSION

The evaluation of the different treatment options differs significantly in the four study groups. The worse the state of health, the more the life‐prolonging effect of cancer therapy is in the foreground, regardless of the adverse events. It is interesting to note that the opinion of the affected physicians can be described as a cross‐section of statements by healthy physicians and melanoma patients. Among all four study groups, a connection can be established between the subjective choice of cancer therapy and the advice given to the patients in the role of the treating physician. However, there is a difference between the individual point of view and the societal point of view regarding the health economic aspect.

## CONFLICT OF INTEREST

JW, MCK and LH have no conflict of interest.

## AUTHOR CONTRIBUTIONS

Conceived and designed the experiments: JW, MCK, and LH. Performed the experiments: JW, MCK, and LH. Analyzed the data: JW, MCK, and LH. Contributed materials/analysis tools: JW, MCK, and LH. Wrote the paper: JW, MCK, and LH. Obtained ethical approval to conduct the questionnaire study: LH.

## Supporting information


**Data S1**

**Data S2**

**Data S3**

## Data Availability

The authors confirm that all data of the study are fully available without restriction. All relevant data are within the paper and its supporting information files.
