# Supplementary material for: Therapy preferences in melanoma treatment—Willingness to pay and preference of quality versus length of life of patients, physicians, healthy individuals and physicians with oncological disease
Source: Cancer Med. 2020 Jul 10;9(17):6132–40. doi: 10.1002/cam4.3191 (PMC7476834; doi:10.1002/cam4.3191)
Supplement: Supplementary file 1 — Data S1 [file CAM4-9-6132-s001.pdf]

Statistically not significant correlations

Group	Parameter 1	Parameter 2
**Healthy individuals**	**Age**	Willingness to sacrifice life years (q. 3)
		"I would agree to a treatment with many side effects (therapy B) at any time, even if the prospect of prolonging my life was very low." (q. 8)
	**Children**	Therapy B versus therapy A (q. 4)
		Therapy B versus therapy C (q. 5)
		Therapy A versus therapy B (q. 6)
		Therapy A versus therapy C (q. 7)
		Cancer therapy versus high sum (q. 18)
		Cancer therapy versus low sum (q. 19)
		"If I were to choose a therapy with high response rates but high rates of side effects (e.g. therapy B), the advice of my family and friends would influence me the most." (q. 10)
		"I would agree to a treatment with many side effects (therapy B) at any time, even if the prospect of prolonging my life was very low." (q. 8)
		Willingness to sacrifice life years (q. 3)
		Willingness to pay for longer therapy intervals (q. 17)
	**Economic status**	Cancer therapy versus high sum (q. 18)
		Cancer therapy versus low sum (q. 19)
		Therapy B versus therapy A (q. 4)
		Therapy B versus therapy C (q. 5)
		Therapy A versus therapy B (q. 6)
		Therapy A versus therapy C (q. 7)
		Willingness to pay for longer therapy intervals (q. 17)
	**Education**	Health policy maker distribution high sum (q. 20)
		Health policy maker distribution low sum (q. 21)
		Willingness to pay for longer therapy intervals (q. 17)
	**Faith**	Therapy B versus therapy A (q. 4)
		Therapy B versus therapy C (q. 5)
		Therapy A versus therapy B (q. 6)
		Therapy A versus therapy C (q. 7)
		"I would agree to a treatment with many side effects (therapy B) at any time, even if the prospect of prolonging my life was very low." (q. 8)
	**Family status**	Therapy B versus therapy A (q. 4)
		Therapy B versus therapy C (q. 5)
		Therapy A versus therapy B (q. 6)
		Therapy A versus therapy C (q. 7)
		Cancer therapy versus high sum (q. 18)
		Cancer therapy versus low sum (q. 19)
		"I would agree to a treatment with many side effects (therapy B) at any time, even if the prospect of prolonging my life was very low." (q. 8)

		Willingness to sacrifice life years (q. 3)
		Willingness to pay for longer therapy intervals (q. 17)
	**Dependents**	Therapy B versus therapy A (q. 4)
		Therapy B versus therapy C (q. 5)
		Therapy A versus therapy B (q. 6)
		Therapy A versus therapy C (q. 7)
		Cancer therapy versus high sum (q. 18)
		Cancer therapy versus low sum (q. 19)
		"If I were to choose a therapy with high response rates but high rates of side effects (e.g. therapy B), the advice of my family and friends would influence me the most." (q. 10)
		"I would agree to a treatment with many side effects (therapy B) at any time, even if the prospect of prolonging my life was very low." (q. 8)
	**Life expectancy**	Willingness to sacrifice life years (q. 3)
	**State of health**	"I would agree to a treatment with many side effects (therapy B) at any time, even if the prospect of prolonging my life was very low." (q. 8)
		"I would prefer early palliative therapy (therapy C) to a therapy rich in side effects if there is no prospect of healing." (q. 11)
		"I would prefer palliative therapy (therapy C) to therapy A or B if my current state of health was poor due to the cancer disease." (q. 9)
		Willingness to sacrifice life years (q. 3)
		"If a treatment could prolong my life, then I would always agree to it, no matter what side effects I had to accept." (q. 12)
**Physicians**	**Age**	"I would agree to a treatment with many side effects (therapy B) at any time, even if the prospect of prolonging my life was very low." (q. 8)
	**Children**	Therapy B versus therapy A (q. 4)
		Therapy B versus therapy C (q. 5)
		Therapy A versus therapy B (q. 6)
		Therapy A versus therapy C (q. 7)
		Cancer therapy versus high sum (q. 18)
		Cancer therapy versus low sum (q. 19)
		"If I were to choose a therapy with high response rates but high rates of side effects (e.g. therapy B), the advice of my family and friends would influence me the most." (q. 10)
		"I would agree to a treatment with many side effects (therapy B) at any time, even if the prospect of prolonging my life was very low." (q. 8)
		Willingness to sacrifice life years (q. 3)
		Willingness to pay for longer therapy intervals (q. 17)
	**Economic status**	Cancer therapy versus high sum (q. 18)
		Cancer therapy versus low sum (q. 19)
		Therapy B versus therapy A (q. 4)
		Therapy B versus therapy C (q. 5)
		Therapy A versus therapy B (q. 6)

		Therapy A versus therapy C (q. 7)
	**Education**	Health policy maker distribution high sum (q. 20)
		Health policy maker distribution low sum (q. 21)
	**Faith**	Therapy B versus therapy C (q. 5)
		Therapy A versus therapy B (q. 6)
		Therapy A versus therapy C (q. 7)
		"I would agree to a treatment with many side effects (therapy B) at any time, even if the prospect of prolonging my life was very low." (q. 8)
	**Family status**	Therapy B versus therapy A (q. 4)
		Therapy B versus therapy C (q. 5)
		Therapy A versus therapy B (q. 6)
		Therapy A versus therapy C (q. 7)
		Cancer therapy versus high sum (q. 18)
		Cancer therapy versus low sum (q. 19)
		"I would agree to a treatment with many side effects (therapy B) at any time, even if the prospect of prolonging my life was very low." (q. 8)
		Willingness to sacrifice life years (q. 3)
		Willingness to pay for longer therapy intervals (q. 17)
	**Dependents**	Therapy B versus therapy A (q. 4)
		Therapy B versus therapy C (q. 5)
		Therapy A versus therapy B (q. 6)
		Therapy A versus therapy C (q. 7)
		Cancer therapy versus high sum (q. 18)
		Cancer therapy versus low sum (q. 19)
		"If I were to choose a therapy with high response rates but high rates of side effects (e.g. therapy B), the advice of my family and friends would influence me the most." (q. 10)
		"I would agree to a treatment with many side effects (therapy B) at any time, even if the prospect of prolonging my life was very low." (q. 8)
	**Life expectancy**	Willingness to sacrifice life years (q. 3)
	**State of health**	"I would agree to a treatment with many side effects (therapy B) at any time, even if the prospect of prolonging my life was very low." (q. 8)
		"I would prefer early palliative therapy (therapy C) to a therapy rich in side effects if there is no prospect of healing." (q. 11)
		"I would prefer palliative therapy (therapy C) to therapy A or B if my current state of health was poor due to the cancer disease." (q. 9)
		"If a treatment could prolong my life, then I would always agree to it, no matter what side effects I had to accept." (q. 12)
**Melanoma patients**	**Age**	Willingness to sacrifice life years (q. 3)
		"I would agree to a treatment with many side effects (therapy B) at any time, even if the prospect of prolonging my life was very low." (q. 8)
	**Children**	Therapy B versus therapy A (q. 4)

		Therapy B versus therapy C (q. 5)
		Therapy A versus therapy B (q. 6)
		Therapy A versus therapy C (q. 7)
		Cancer therapy versus high sum (q. 18)
		Cancer therapy versus low sum (q. 19)
		"If I were to choose a therapy with high response rates but high rates of side effects (e.g. therapy B), the advice of my family and friends would influence me the most." (q. 10)
		"I would agree to a treatment with many side effects (therapy B) at any time, even if the prospect of prolonging my life was very low." (q. 8)
		Willingness to sacrifice life years (q. 3)
		Willingness to pay for longer therapy intervals (q. 17)
	**Economic status**	Cancer therapy versus high sum (q. 18)
		Cancer therapy versus low sum (q. 19)
		Therapy B versus therapy A (q. 4)
		Therapy B versus therapy C (q. 5)
		Therapy A versus therapy B (q. 6)
		Therapy A versus therapy C (q. 7)
	**Education**	Health policy maker distribution high sum (q. 20)
		Health policy maker distribution low sum (q. 21)
		Willingness to pay for longer therapy intervals (q. 17)
	**Faith**	Therapy B versus therapy A (q. 4)
		Therapy B versus therapy C (q. 5)
		Therapy A versus therapy B (q. 6)
		Therapy A versus therapy C (q. 7)
		"I would agree to a treatment with many side effects (therapy B) at any time, even if the prospect of prolonging my life was very low." (q. 8)
	**Family status**	Therapy B versus therapy A (q. 4)
		Therapy B versus therapy C (q. 5)
		Therapy A versus therapy B (q. 6)
		Therapy A versus therapy C (q. 7)
		Cancer therapy versus high sum (q. 18)
		Cancer therapy versus low sum (q. 19)
		"I would agree to a treatment with many side effects (therapy B) at any time, even if the prospect of prolonging my life was very low." (q. 8)
		Willingness to sacrifice life years (q. 3)
		Willingness to pay for longer therapy intervals (q. 17)
	**Dependents**	Therapy B versus therapy A (q. 4)
		Therapy B versus therapy C (q. 5)
		Therapy A versus therapy B (q. 6)
		Therapy A versus therapy C (q. 7)
		Cancer therapy versus high sum (q. 18)
		Cancer therapy versus low sum (q. 19)

		"If I were to choose a therapy with high response rates but high rates of side effects (e.g. therapy B), the advice of my family and friends would influence me the most." (q. 10)
		"I would agree to a treatment with many side effects (therapy B) at any time, even if the prospect of prolonging my life was very low." (q. 8)
	**Life expectancy**	Willingness to sacrifice life years (q. 3)
	**State of health**	"I would agree to a treatment with many side effects (therapy B) at any time, even if the prospect of prolonging my life was very low." (q. 8)
		"I would prefer early palliative therapy (therapy C) to a therapy rich in side effects if there is no prospect of healing." (q. 11)
		"I would prefer palliative therapy (therapy C) to therapy A or B if my current state of health was poor due to the cancer disease." (q. 9)
		Willingness to sacrifice life years (q. 3)
		"If a treatment could prolong my life, then I would always agree to it, no matter what side effects I had to accept." (q. 12)
**Physicians with oncological disease**	**Age**	Willingness to sacrifice life years (q. 3)
		"I would agree to a treatment with many side effects (therapy B) at any time, even if the prospect of prolonging my life was very low." (q. 8)
	**Children**	Therapy B versus therapy A (q. 4)
		Therapy B versus therapy C (q. 5)
		Therapy A versus therapy B (q. 6)
		Therapy A versus therapy C (q. 7)
		Cancer therapy versus high sum (q. 18)
		Cancer therapy versus low sum (q. 19)
		"I would agree to a treatment with many side effects (therapy B) at any time, even if the prospect of prolonging my life was very low." (q. 8)
		Willingness to sacrifice life years (q. 3)
		Willingness to pay for longer therapy intervals (q. 17)
	**Economic status**	Cancer therapy versus high sum (q. 18)
		Cancer therapy versus low sum (q. 19)
		Therapy B versus therapy A (q. 4)
		Therapy B versus therapy C (q. 5)
		Therapy A versus therapy B (q. 6)
		Therapy A versus therapy C (q. 7)
		Willingness to pay for longer therapy intervals (q. 17)
	**Education**	Health policy maker distribution high sum (q. 20)
		Health policy maker distribution low sum (q. 21)
	**Faith**	Therapy B versus therapy A (q. 4)
		Therapy B versus therapy C (q. 5)
		Therapy A versus therapy B (q. 6)

		"I would agree to a treatment with many side effects (therapy B) at any time, even if the prospect of prolonging my life was very low." (q. 8)
	**Family status**	Therapy B versus therapy A (q. 4)
		Therapy B versus therapy C (q. 5)
		Therapy A versus therapy B (q. 6)
		Therapy A versus therapy C (q. 7)
		Cancer therapy versus high sum (q. 18)
		Cancer therapy versus low sum (q. 19)
		"I would agree to a treatment with many side effects (therapy B) at any time, even if the prospect of prolonging my life was very low." (q. 8)
		Willingness to sacrifice life years (q. 3)
		Willingness to pay for longer therapy intervals (q. 17)
	**Dependents**	Therapy B versus therapy A (q. 4)
		Therapy B versus therapy C (q. 5)
		Therapy A versus therapy B (q. 6)
		Therapy A versus therapy C (q. 7)
		Cancer therapy versus high sum (q. 18)
		Cancer therapy versus low sum (q. 19)
		"If I were to choose a therapy with high response rates but high rates of side effects (e.g. therapy B), the advice of my family and friends would influence me the most." (q. 10)
	**Life expectancy**	Willingness to sacrifice life years (q. 3)
	**State of health**	"I would agree to a treatment with many side effects (therapy B) at any time, even if the prospect of prolonging my life was very low." (q. 8)
		"I would prefer early palliative therapy (therapy C) to a therapy rich in side effects if there is no prospect of healing." (q. 11)
		"I would prefer palliative therapy (therapy C) to therapy A or B if my current state of health was poor due to the cancer disease." (q. 9)
		Willingness to sacrifice life years (q. 3)
		"If a treatment could prolong my life, then I would always agree to it, no matter what side effects I had to accept." (q. 12)
**All groups**	**Age**	"I would recommend early palliative therapy (therapy C)." (q. 25)
	**Children**	"I would point out palliative therapy possibilities (therapy C) already at the time of diagnosis." (q. 29)
		"Since no cure is possible in the final stage of cancer, I would rather try to improve the quality of life than fight the tumor." (q. 31)
		"I would recommend early palliative therapy (therapy C)." (q. 25)
		Willingness to sacrifice life years (q. 3)
		Therapy A versus therapy C (q. 7)
		Therapy B versus therapy A (q. 4)
		Therapy A versus therapy B (q. 6)

		"I would agree to a treatment with many side effects (therapy B) at any time, even if the prospect of prolonging my life was very low." (q. 8)
		"If a treatment could prolong my life, then I would always agree to it, no matter what side effects I had to accept." (q. 12)
		"I would always choose a therapy that has the best chance of prolonging life, even though the side effects can be severe to life-threatening." (q. 15)
		"I would rather make the best of my remaining months of life (e.g. travel, visit family and friends) than undergo a stressful therapy (therapy A), which can only prolong my life by months despite serious side effects." (q. 16)
		"If I were to choose a therapy with high response rates but high rates of side effects (e.g. therapy B), the advice of my family and friends would influence me the most." (q. 10)
		Willingness to sacrifice life years (q. 3)
		"If a treatment could prolong my life, then I would always agree to it, no matter what side effects I had to accept." (q. 12)
	**Economic status**	Therapy B versus therapy A (q. 4)
		Therapy A versus therapy B (q. 6)
		Cancer therapy versus high sum (q. 18)
		Cancer therapy versus low sum (q. 19)
	**Education**	"If I should decide on a therapy with high response rates but high side effect rates (e.g. therapy B), the advice of my attending physician would influence me most." (q. 14)
	**Faith**	Therapy B versus therapy A (q. 4)
		Therapy B versus therapy C (q. 5)
		Therapy A versus therapy B (q. 6)
		Therapy A versus therapy C (q. 7)
		"I would agree to a treatment with many side effects (therapy B) at any time, even if the prospect of prolonging my life was very low." (q. 8)
		"If I were to choose a therapy with high response rates but high rates of side effects (e.g. therapy B), the advice of my family and friends would influence me the most." (q. 10)
		Willingness to sacrifice life years (q. 3)
	**Family status**	"I would point out palliative therapy possibilities (therapy C) already at the time of diagnosis." (q. 29)
		"Since no cure is possible in the final stage of cancer, I would rather try to improve the quality of life than fight the tumor." (q. 31)
		"I would recommend early palliative therapy (therapy C)." (q. 25)
		Willingness to sacrifice life years (q. 3)
		Therapy B versus therapy A (q. 4)
		Therapy B versus therapy C (q. 5)
		Therapy A versus therapy B (q. 6)
		Therapy A versus therapy C (q. 7)
		"I would agree to a treatment with many side effects (therapy B) at any time, even if the prospect of prolonging my life was very low." (q. 8)
		"I would always choose a therapy that has the best chance of prolonging life, even though the side effects can be severe to life-threatening." (q. 15)

		I would rather make the best of my remaining months of life (e.g. travel, visit family and friends) than undergo a stressful therapy (therapy A), which can only prolong my life by months despite serious side effects.
	**Dependents**	Therapy B versus therapy A (q. 4)
		Therapy B versus therapy C (q. 5)
		Therapy A versus therapy B (q. 6)
		Therapy A versus therapy C (q. 7)
		"I would always choose a therapy that has the best chance of prolonging life, even though the side effects can be severe to life-threatening." (q. 15)
	**State of health**	"I would point out palliative therapy possibilities (therapy C) already at the time of diagnosis." (q. 29)
		"Since no cure is possible in the final stage of cancer, I would rather try to improve the quality of life than fight the tumor." (q. 31)
