# Supplementary material for: Therapy preferences in melanoma treatment—Willingness to pay and preference of quality versus length of life of patients, physicians, healthy individuals and physicians with oncological disease
Source: Cancer Med. 2020 Jul 10;9(17):6132–40. doi: 10.1002/cam4.3191 (PMC7476834; doi:10.1002/cam4.3191)
Supplement: Supplementary file 2 — Data S2 [file CAM4-9-6132-s002.pdf]

Study questionnaire "Cancer therapies and their evaluation"

This questionnaire examines how therapeutic decisions are made between different cancer therapies. The survey is anonymous. We would appreciate if you would take the time to answer our questions. It takes about 20 minutes to complete the questionnaire. Please answer **all** questions. The following questions are purely hypothetical and have nothing to do with your therapy.

1) Current state of health on a scale of 0-100%

Please indicate on the line how you feel at the moment.

Worst conceivable state of health	0%	10%	20%	30%	40%	50%	60%	70%	80%	90%	100%	Best conceivable state of health

----

Estimated life expectancy

2) I think I will be ___________ years old.

Let's assume your actual life expectancy is 80 years and you would have to struggle with daily complaints such as back pain or visual and hearing loss until then. Imagine that you could live without these complaints for the rest of your life, but would have to sacrifice years of your life in return. Your life is so shortened, but you have no health problems. How many years of life would you be willing to sacrifice?

3) I would be willing to sacrifice ____________ years of my life if I could live for the rest of my life without any health problems.

Please put yourself in this situation:

You suffer from advanced cancer that has already spread to internal organs. You will be informed that you are suffering from an incurable disease and the average life expectancy without treatment is 9 months. There is a choice between four treatment options (table below):

Therapy A (standard immunotherapy)

There is a therapy (infusion) to which **40%** of patients respond, i.e. the tumour disappears or becomes smaller. Patients live **on average 11 months** longer with this drug. Side effects include diarrhoea, nausea, hormonal imbalances, skin rash, fatigue and liver dysfunction. About **15%** of patients experience **severe to life-threatening side effects**. The costs are about **70'00€.**

Therapy B (combination immunotherapy)

With an extended therapy (infusion) **50%** of patients respond, i.e. the tumour disappears or becomes smaller. Patients live **on average 21 months** longer with this drug. Side effects include diarrhoea, nausea, hormonal imbalances, skin rash, fatigue and liver dysfunction. Serious to life-threatening side effects occur in about **36%** of patients. The costs are about **150'000€.**

Therapy C (palliative therapy)

Palliative care does not include any specific therapy for the tumor disease. The focus is on pain relief and relief from symptoms that arise as a result of cancer. Patients receive psychological and, if desired, spiritual support and are advised and supported in medical, financial and social matters. Through the cooperation of nurses, doctors, social workers and clergymen, a more personalized care is made possible. The costs are **4'900 €** per patient per year.

Tabelle:

**Therapy**	**Response rate = proportion of patients, in which the drug is effective**	**Average life extension**	**Serious side effects**	**Costs (pays health insurance)**
**A standard immunotherapy**	40%	11 months	15%	70'000 €
**B combination immunotherapy**	50%	21 months	36%	150'000 €
**C palliative therapy**	No response rate, since this form of therapy alleviates complaints and pain	none	none	4'900 €

For each pair of treatment options below, please select which situation you would prefer in each case.

You have to decide anew for each pair of therapy options.

4) ☐ **Either** 19 months living with therapy B and severe side effects in 36%

☐ **or** 9 months living with therapy A and severe side effects in 15%

5) ☐ **Either** 24 months living with therapy B and severe side effects in 36%

☐ **or** 3 months of pain-free living without tumor therapy with palliative therapy (therapy C)

6) ☐ **Either** 12 months living with therapy B and severe side effects in 36%

☐ **or** 11 months living with therapy A and severe side effects in 15%

7) ☐ **Either** 9 months living with therapy A and severe side effects in 15%

☐ **or** 3 months of pain-free living without tumor therapy with palliative therapy (therapy C)

<u>Personal therapy decision</u>

*<u>Please consider **for each individual statement** to what extent you agree and tick only one option per statement.</u>*

	I absolutely agree	I agree	I am undecided	I disagree	I absolutely disagree
**8)** I would agree to a treatment with many side effects (therapy B) at any time, even if the prospect of prolonging my life was very low.	☐	☐	☐	☐	☐
**9)** I would prefer palliative therapy (therapy C) to therapy A or B if my current state of health was poor due to the cancer disease.	☐	☐	☐	☐	☐
**10)** If I were to choose a therapy with high response rates but high rates of side effects (e.g. therapy B), the advice of my family and friends would influence me the most.	☐	☐	☐	☐	☐
**11)** I would prefer early palliative therapy (therapy C) to a therapy rich in side effects if there is no prospect of healing.	☐	☐	☐	☐	☐
**12)** If a treatment could prolong my life, then I would always agree to it, no matter what side effects I had to accept.	☐	☐	☐	☐	☐

	I absolutely agree	I agree	I am undecided	I disagree	I absolutely disagree
**13)** I would prefer to receive the infusions every three weeks rather than every two weeks if the effect is equivalent.	☐	☐	☐	☐	☐
**14)** If I should decide on a therapy with high response rates but high side effect rates (e.g. therapy B), the advice of my attending physician would influence me most.	☐	☐	☐	☐	☐
**15)** I would always choose a therapy that has the best chance of prolonging life, even though the side effects can be severe to life-threatening.	☐	☐	☐	☐	☐
**16)** I would rather make the best of my remaining months of life (e.g. travel, visit family and friends) than undergo a stressful therapy (therapy A), which can only prolong my life by months despite serious side effects.	☐	☐	☐	☐	☐

17) How much money would you be willing to pay if you had to come to the clinic for therapy only every three weeks instead of every two weeks?

☐ None ☐ 200 € ☐ 400 € ☐ 1000€ ☐ ___________ (sum)

18) If you **yourself** were to receive 150'000 € for your therapy and were free to decide how to spend it, how would you use this money?

☐ A) I would opt for the new combination immunotherapy (therapy B).

☐ B) I would opt for the standard immunotherapy (therapy A) and want to have the difference **(80'000 €)** in cash at my free disposal.

☐ C) I would renounce any tumor therapy and prefer the whole amount **(150'000 €)** in cash to fulfill a last wish or not to have financial worries.

19) Please choose between the following options:

☐ A) I would opt for the new combination immunotherapy (therapy B).

☐ B) I would opt for the standard immunotherapy (therapy A) and want to have **40’000 €** in cash at my free disposal.

☐ C) I **do not want** tumor therapy and **80'000 €** cash at my free disposal.

Now please assume the role of a health policy decision-maker (e.g. health insurance company)

The pharmaceutical industry has set costs of approximately 150'000 € per treatment cycle for therapy B (combination immunotherapy). The financial resources of the health insurance companies are limited, so in everyday clinical practice it must always be individually assessed in which case the new medications should be used.

Please tick one of the possible answers in the following selection questions.

20) If **you** were authorised to decide on the investment of €1.5 million from the health fund, which decision would you take?

☐ A) I enable 306 patients to be cared for by palliative therapy (therapy C), whereby a better quality of life, but not a prolonged life can be achieved.

☐ B) I treat 10 patients with therapy B and thus enable them to survive 21 months longer on average.

21) If **you** had to distribute 150'000 € from our health system, how would you decide?

☐ A) I invest it for one patient in a treatment cycle with the new combination immunotherapy (therapy B).

☐ B) I enable palliative therapy (therapy C) for 30 patients for one year.

☐ C) I enable 11'252 skin screenings to detect about 42 cases of melanoma at an early stage, where healing is possible.

<u>Now please put yourself in the role of the treating physician.</u>

<u>Please read the following statements about the treatment decision and tick only one option per statement.</u>

	**I absolutely agree**	**I agree**	**I am undecided**	**I disagree**	**I absolutely disagree**
**22)** Combination immunotherapy (therapy B) is a real advance over standard immunotherapy (therapy A).	☐	☐	☐	☐	☐
**23)** I would not use combination immunotherapy (therapy B) because the probability of side effects and the gain in life extension are disproportionate.	☐	☐	☐	☐	☐
**24)** I would always recommend a treatment with more side effects (therapy B) compared to a better tolerated therapy (therapy A), even if the prospect of an additional prolongation of life would be minimal.	☐	☐	☐	☐	☐
**25)** I would recommend early palliative therapy (therapy C).	☐	☐	☐	☐	☐
**26)** I would always start the treatment of a patient with standard immunotherapy (therapy A) before using combination immunotherapy (therapy B).	☐	☐	☐	☐	☐

	I absolutely agree	I agree	I am undecided	I disagree	I absolutely disagree
**27)** I would be rather reluctant to use combination immunotherapy (therapy B), because otherwise a lot of money would be consumed, which could be used for research for example.	☐	☐	☐	☐	☐
**28)** I would dissuade my patients from combination immunotherapy (therapy B).	☐	☐	☐	☐	☐
**29)** I would point out palliative therapy possibilities (therapy C) already at the time of diagnosis.	☐	☐	☐	☐	☐
**30)** I would be rather reluctant to use the combination immunotherapy (therapy B), because otherwise a lot of money is consumed, which could be used for example for prevention measures against cancer.	☐	☐	☐	☐	☐
**31)** Since no cure is possible in the final stage of cancer, I would rather try to improve the quality of life than fight the tumor.	☐	☐	☐	☐	☐

Personal data:

32) Age ______

33) Gender ☐ Male ☐ Female

34) Family status:

☐ alone ☐ with partner ☐ with partner and child ☐ single parent ☐ with others

35) Do you have **children**? ☐ Yes ☐ No

36) Do you have someone who lives **dependent** on you (e.g. children, foster care)? ☐ Yes (who) ______________________________ ☐ No

37) How important is **religious belief** for you in everyday life:

☐ high ☐ medium ☐ little ☐ none ☐ no statement

38) Please mark with a cross which **education** you have.

☐ none ☐ apprenticeship ☐ master/ technical college degree ☐ university degree ☐ __________

39) What is your **employment** relationship?

☐ employee ☐ self-employed ☐ Other

40) Please tick the box in the right-hand column indicating the range of your current **monthly gross income**.

Less than 500 Euro per month	
500 – 1'000 € per month	
1'000 – 2'000 € per month	
2'000 – 3'500 € per month	
3'500 – 5'000 € per month	
5'000 € and more per month	

We thank you very much for your participation in our study and for your time.
