# Supplementary material for: Therapy preferences in melanoma treatment—Willingness to pay and preference of quality versus length of life of patients, physicians, healthy individuals and physicians with oncological disease
Source: Cancer Med. 2020 Jul 10;9(17):6132–40. doi: 10.1002/cam4.3191 (PMC7476834; doi:10.1002/cam4.3191)
Supplement: Supplementary file 3 — Data S3 [file CAM4-9-6132-s003.pdf]

Fragebogen zur Studie „Krebstherapien und ihre Bewertung“

Dieser Fragebogen untersucht wie Therapieentscheidungen zwischen verschiedenen Krebstherapien getroffen werden. Die Befragung ist anonym. Wir würden uns freuen, wenn Sie sich Zeit nehmen würden, unsere Fragen zu beantworten. Die Bearbeitung des Fragebogens dauert etwa 20 Minuten. Bitte beantworten Sie **alle** Fragen. Die folgenden Fragen sind rein hypothetisch und haben **nichts** mir **Ihrer** Therapie zu tun.

1) Aktueller Gesundheitszustand auf einer Skala von 0-100%

Bitte geben Sie auf der Linie an, wie Sie sich gerade im Moment fühlen.

Schlechtester vorstellbarer Gesundheitszustand	0%	10%	20%	30%	40%	50%	60%	70%	80%	90%	100%	Bester vorstellbarer Gesundheitszustand

----

Geschätzte Lebenserwartung

2) Ich denke, ich werde ___________Jahre alt.

Angenommen ihre tatsächliche Lebenserwartung liegt bei 80 Jahren und Sie hätten bis dahin mit täglichen Beschwerden wie Rückenschmerzen oder Seh- und Hörminderung zu kämpfen. Stellen Sie sich vor, Sie könnten bis an Ihr Lebensende ohne diese Beschwerden leben, müssten dafür aber im Gegenzug Lebensjahre opfern. Ihr Leben ist so verkürzt, haben aber keine gesundheitlichen Probleme. Wie viele Lebensjahre wären Sie bereit zu opfern?

3) Ich wäre bereit ____________ Lebensjahre zu opfern, wenn ich dafür bis an mein Lebensende ohne jegliche gesundheitliche Beschwerden leben könnte.

Versetzen Sie sich bitte in diese Situation:

Sie leiden an fortgeschrittenem Krebs, der schon in innere Organe gestreut hat. Sie werden darüber aufgeklärt, dass Sie an einer unheilbaren Krankheit leiden und die durchschnittliche Lebenserwartung ohne Behandlung 9 Monate sei. Es gibt vier Therapieoptionen (Tabelle unten):

Therapie A (Standard-Immuntherapie)

Es gibt eine Therapie (Infusion), auf die **40%** der Patienten ansprechen, d.h. der Tumor verschwindet oder wird kleiner. Patienten leben mit diesem Medikament im **Durchschnitt 11 Monate** länger. Die Nebenwirkungen umfassen Durchfall, Übelkeit, Hormonstörungen, Hautausschlag, Müdigkeit und Leberfunktionsstörungen. Bei ca. **15%** der Patienten treten **schwerwiegende bis lebensbedrohende Nebenwirkungen** auf. Die Kosten liegen bei **70.000€**.

Therapie B (Kombinations-Immuntherapie)

Bei einer erweiterten Therapie (Infusion) sprechen **50%** der Patienten an, d.h. der Tumor verschwindet oder wird kleiner. Patienten leben mit diesem Medikament im **Durchschnitt 21 Monate** länger. Die Nebenwirkungen umfassen Durchfall, Übelkeit, Hormonstörungen, Hautausschlag, Müdigkeit und Leberfunktionsstörungen. Bei ca. **36%** der Patienten treten **schwerwiegende bis lebensbedrohende Nebenwirkungen** auf. Die Kosten liegen bei **150.000€**.

Therapie C (Palliativtherapie)

Die Palliativversorgung umfasst keine spezifische Therapie der Tumorerkrankung. Im Mittelpunkt stehen Schmerzlinderung und Befreiung von Beschwerden, welche als Folgen der Krebserkrankung entstehen. Patienten erhalten psychologischen und auf Wunsch auch geistlichen Beistand und werden in medizinischen, finanziellen und sozialen Fragen beraten und unterstützt. Durch die Zusammenarbeit von Krankenschwestern, Ärzten, Sozialarbeitern und Geistlichen wird eine persönlichere Betreuung ermöglicht. Die Kosten liegen bei **4.900 €** pro Patient pro Jahr.

Tabelle:

**Therapie**	**Ansprechrate = Anteil von Patienten, bei denen das Medikament wirkt**	**Durchschnittliche Lebensverlängerung**	**Schwerwiegende Nebenwirkungen**	**Kosten (zahlt Krankenkasse)**
**A Standard-Immuntherapie**	40%	11 Monate	15%	70.000 €
**B Kombinations-Immuntherapie**	50%	21 Monate	36%	150.000 €
**C Palliativtherapie**	Keine Ansprechrate, da bei dieser Therapieform Beschwerden und Schmerzen gelindert werden	keine	keine	4.900 €

<u>Treffen Sie bitte bei jedem Paar der unten gegenübergestellten Therapieoptionen eine Auswahl, welche Situation Sie im jeweiligen Fall vorziehen würden.</u>

<u>Sie müssen sich bei jedem Paar von Therapieoptionen neu entscheiden.</u>

4) ☐ **Entweder** 19 Monate leben mit Therapie B und schweren Nebenwirkungen in 36%

☐ **oder** 9 Monate leben mit Therapie A und schweren Nebenwirkungen in 15%

5) ☐ **Entweder** 24 Monate leben mit Therapie B und schweren Nebenwirkungen in 36%

☐ **oder** 3 Monate beschwerdefrei leben ohne Tumortherapie mit palliativmedizinischer Betreuung (Therapie C)

6) ☐ **Entweder** 12 Monate leben mit Therapie B und schweren Nebenwirkungen in 36%

☐ **oder** 11 Monate leben mit Therapie A und schweren Nebenwirkungen in 15%

7) ☐ **Entweder** 9 Monate leben mit Therapie A und schweren Nebenwirkungen in 15%

☐ **oder** 3 Monate beschwerdefrei leben ohne Tumortherapie mit palliativmedizinischer Betreuung (Therapie C)

<u>Persönliche Therapieentscheidung</u>

*<u>Bitte überlegen Sie **für jede einzelne Aussage** in wieweit Sie zustimmen und kreuzen Sie pro Aussage bitte nur **eine** Möglichkeit an.</u>*

	**Ich stimme voll zu**	**Ich stimme eher zu**	**Ich bin unentschlossen**	**Ich stimme eher nicht zu**	**Ich stimme überhaupt nicht zu**
**8)** Ich würde einer nebenwirkungsreichen Behandlung (Therapie B) jederzeit zustimmen, auch wenn die Aussicht auf eine Lebensverlängerung nur sehr gering wäre.	☐	☐	☐	☐	☐
**9)** Ich würde eine palliativmedizinische Behandlung (Therapie C) der Therapie A oder B vorziehen, wenn mein aktueller Gesundheitszustand aufgrund der Krebserkrankung schlecht wäre.	☐	☐	☐	☐	☐
**10)** Wenn ich mich für eine Therapie mit hohen Ansprechraten, aber hohen Nebenwirkungsraten (z.B. Therapie B) entscheiden sollte, würde mich der Rat meiner Familie und meiner Freunde am meisten beeinflussen.	☐	☐	☐	☐	☐
**11)** Ich würde die frühzeitige palliativmedizinische Behandlung (Therapie C) gegenüber einer nebenwirkungsreichen Therapie vorziehen, wenn keine Aussicht auf Heilung besteht.	☐	☐	☐	☐	☐
**12)** Wenn eine Behandlung mein Leben verlängern könnte, dann würde ich dieser immer zustimmen, egal welche Nebenwirkungen ich in Kauf nehmen müsste.	☐	☐	☐	☐	☐

	Ich stimme voll zu	Ich stimme eher zu	Ich bin unentschlossen	Ich stimme eher nicht zu	Ich stimme überhaupt nicht zu
**13)** Bei gleichwertiger Wirkung möchte ich die Infusionen lieber alle drei Wochen als alle zwei Wochen bekommen.	☐	☐	☐	☐	☐
**14)** Wenn ich mich für eine Therapie mit hohen Ansprechraten, aber hohen Nebenwirkungsraten (z.B. Therapie B) entscheiden sollte, würde mich der Rat meines behandelnden Arztes am meisten beeinflussen.	☐	☐	☐	☐	☐
**15)** Ich würde mich immer für eine Therapie entscheiden, welche die besten Aussichten auf eine Lebensverlängerung besitzt, auch wenn die Nebenwirkungen schwerwiegend bis lebensbedrohend sein können.	☐	☐	☐	☐	☐
**16)** Ich würde lieber das Beste aus meinen verbleibenden Lebensmonaten machen (z.B. Reisen, Familie, Freunde besuchen) als mich einer belastenden Therapie (Therapie A) zu unterziehen, die trotz schwerwiegender Nebenwirkungen mein Leben nur um Monate verlängern kann.	☐	☐	☐	☐	☐

17) Wie viel Geld wären Sie privat <u>maximal</u> bereit zu zahlen, wenn Sie für die Therapie nur alle drei Wochen statt alle zwei Wochen in die Klinik kommen müssten?

☐ Keines ☐ 200 € ☐ 400 € ☐ 1000€ ☐ __________ (Betrag)

18) Wenn Sie **selbst** 150.000 € für Ihre Therapie bekämen und frei entscheiden können wofür sie es ausgeben, wie würden Sie dieses Geld einsetzen?

☐ A) Ich würde mich für die neue Kombinations-Immuntherapie (Therapie B) entscheiden.

☐ B) Ich würde mich für die Standard-Immuntherapie (Therapie A) entscheiden und die Differenz (**80.000 €**) in bar zur freien Verfügung haben wollen.

☐ C) Ich würde auf jede Tumortherapie verzichten und die ganze Summe (**150.000 €**) in bar vorziehen, um mir einen letzten Wunsch zu erfüllen oder keine finanziellen Sorgen haben zu müssen.

19) Wählen Sie zwischen folgenden Möglichkeiten:

☐ A) Ich möchte die Kombinations-Immuntherapie (Therapie B) erhalten.

☐ B) Ich möchte die Standard-Immuntherapie (Therapie A) und **40.000 €** in bar zur freien Verfügung.

☐ C) Ich möchte **<u>keine</u>** Tumortherapie und **80.000 €** in bar zur freien Verfügung.

<u>Übernehmen Sie nun bitte die Rolle eines gesundheitspolitischen Entscheidungsträgers (z.B. Krankenkasse)</u>

Die pharmazeutische Industrie hat für Therapie B (Kombinations-Immuntherapie) Kosten von ca. 150.000 € pro Behandlungszyklus festgesetzt. Die finanziellen Mittel der Krankenkassen sind begrenzt, daher muss im klinischen Alltag immer wieder individuell abgewogen werden, in welchem Fall die neuen Präparate eingesetzt werden sollen.

<u>Kreuzen Sie bitte in den folgenden Auswahlfragen jeweils eine der Antwortmöglichkeiten an.</u>

20) Wenn **Sie** befugt wären, über die Investition von 1,5 Mio. € aus dem Gesundheitsfond zu entscheiden, welche Entscheidung würden Sie treffen?

☐ A) Ich ermögliche 306 Patienten die Betreuung durch ein palliativmedizinisches Team (Therapie C) wodurch eine bessere Lebensqualität, aber kein verlängertes Leben erreicht werden kann.

☐ B) Ich behandle 10 Patienten mit Therapie B und ermögliche diesen somit im Durchschnitt ein 21 Monate längeres Überleben.

21) Wenn **Sie** 150.000 € aus unserem Gesundheitssystem verteilen müssten, wie würden Sie sich entscheiden?

☐ A) Ich investiere sie für <u>einen</u> Patienten in <u>einen</u> Behandlungszyklus mit der neuen Kombinations-Immuntherapie (Therapie B).

☐ B) Ich ermögliche eine Palliativbehandlung (Therapie C) für 30 Patienten für ein Jahr.

☐ C) Ich ermögliche 11.252 Hautscreening-Untersuchungen, um etwa 42 Fälle von schwarzem Hautkrebs früh zu entdecken, wo Heilung möglich ist.

<u>Versetzen Sie sich nun bitte in die Situation des behandelnden Arztes.</u>

<u>Bitte lesen Sie die folgenden Aussagen über die Behandlungsentscheidung durch und kreuzen Sie pro Aussage bitte nur eine Möglichkeit an.</u>

	**Ich stimme voll zu**	**Ich stimme eher zu**	**Ich bin unentschlossen**	**Ich stimme eher nicht zu**	**Ich stimme überhaupt nicht zu**
**22)** Die Kombinations-Immuntherapie (Therapie B) ist gegenüber der Standard-Immuntherapie (Therapie A) ein echter Fortschritt.	☐	☐	☐	☐	☐
**23)** Ich würde die Kombinations-Immuntherapie (Therapie B) nicht einsetzen, da die Wahrscheinlichkeit von Nebenwirkungen und der Zugewinn an Lebensverlängerung in keinem Verhältnis stehen.	☐	☐	☐	☐	☐
**24)** Ich würde eine nebenwirkungsreichere Behandlung (Therapie B) gegenüber einer besser verträglichen Therapie (Therapie A) jederzeit empfehlen, auch wenn die Aussicht auf eine zusätzliche Lebensverlängerung nur gering wäre.	☐	☐	☐	☐	☐
**25)** Ich würde eine frühzeitige palliativmedizinische Behandlung (Therapie C) empfehlen.	☐	☐	☐	☐	☐
**26)** Ich würde die Behandlung eines Patienten in jedem Fall mit der Standard-Immuntherapie (Therapie A) beginnen, bevor ich die Kombinations-Immuntherapie (Therapie B) einsetzen würde.	☐	☐	☐	☐	☐

	**Ich stimme voll zu**	**Ich stimme eher zu**	**Ich bin unentschlossen**	**Ich stimme eher nicht zu**	**Ich stimme überhaupt nicht zu**
**27)** Ich wäre mit der Kombinations-Immuntherapie (Therapie B) eher zurückhaltend, da sonst viel Geld verbraucht wird, welches man beispielsweise für Forschung nutzen könnte.	☐	☐	☐	☐	☐
**28)** Ich würde meinen Patienten von der Kombinations-Immuntherapie (Therapie B) abraten.	☐	☐	☐	☐	☐
**29)** Ich würde auf die palliativmedizinischen Möglichkeiten (Therapie C) schon bei der Diagnose hinweisen.	☐	☐	☐	☐	☐
**30)** Ich wäre mit der Kombinations-Immuntherapie (Therapie B) eher zurückhaltend, da sonst viel Geld verbraucht wird, welches man beispielsweise für Präventionsmaßnahmen gegen Krebs nutzen könnte.	☐	☐	☐	☐	☐
**31)** Da im Endstadium einer Krebserkrankung keine Heilung mehr möglich ist, würde ich eher versuchen die Lebensqualität zu verbessern, als den Tumor zu bekämpfen.	☐	☐	☐	☐	☐

Angaben zur Person:

32) Alter ______

33) Geschlecht ☐ Männlich ☐ Weiblich

34) Familienstand:

☐ allein lebend ☐ mit Partner/in lebend ☐ mit Partner und Kind lebend ☐ allein erziehend ☐ mit anderen Personen lebend

35) Haben Sie Kinder? ☐ Ja ☐ Nein

36) Haben Sie jemanden, der abhängig von Ihnen lebt (z.B. Kinder, Pflegefall): ☐ Ja (wen) ____ ☐ Nein

37) Wie wichtig ist der **religiöse Glaube** für Sie im Alltag:

☐ sehr ☐ mittel ☐ wenig ☐ gar nicht ☐ keine Angabe

38) Bitte kreuzen Sie an, welche **Ausbildung** Sie haben.

☐ Keine ☐ Lehre ☐ Meister/Fachschule ☐ Hochschulabschluss ☐ __________________________

39) Wie ist Ihr **Arbeitsverhältnis**?

☐ Angestellt ☐ Selbstständig ☐ Anderes (z.B. Arbeitslosigkeit, Rente, Studium)

40) Bitte kreuzen Sie in der rechten Spalte an, in welchem Bereich die **Höhe Ihres aktuellen monatlichen Bruttoeinkommens** liegt.

unter 500 Euro pro Monat	
500 – 1.000 Euro pro Monat	
1.000 – 2.000 Euro pro Monat	
2.000 – 3.500 Euro pro Monat	
3.500 – 5.000 Euro pro Monat	
5.000 und mehr Euro pro Monat	

Wir bedanken uns recht herzlich für Ihre Teilnahme an unserer Studie und für Ihren zeitlichen Aufwand.
